# Ostraceous hyperkeratotic plaques on the extremities: A case of severe hypertrophic lichen planus previously misdiagnosed as atopic dermatitis

**DOI:** 10.1016/j.jdcr.2025.08.014

**Published:** 2025-08-29

**Authors:** Grant J. Riew, Michael S. Chang, Kevin Fettel, Alvaro C. Laga, Ryan Chen, Vinod E. Nambudiri

**Affiliations:** a Harvard Medical School, Boston, Massachusetts; b Department of Dermatology, Brigham and Women’s Hospital, Boston, Massachusetts; c Department of Pathology, Brigham and Women’s Hospital, Boston, Massachusetts; d University of Massachusetts Chan Medical School, Worcester, Massachusetts

**Keywords:** hypertrophic lichen planus, lichen planus

## Case description

A 77-year-old woman with a past medical history significant for venous insufficiency and hypothyroidism presented with a 1-year history of progressively worsening, intensely painful skin lesions. Initially, lesions appeared as thin, pink, scaly plaques predominantly on her lower legs, which evolved into thick, yellow, horn-like crusted keratotic projections associated with severe pain on palpation. The patient was initially diagnosed with atopic dermatitis by 2 independent dermatologists at the onset of the thin pink lesions and initiated on multiple treatments, including potent topical corticosteroids, a topical Janus kinase (JAK) inhibitor, and systemic dupilumab, which failed to halt disease progression. Additional treatment with doxycycline and cefalexin for suspected secondary bacterial infection provided no clinical improvement. Due to disease progression, she presented to our institution for repeat workup.

**Fig 1:**
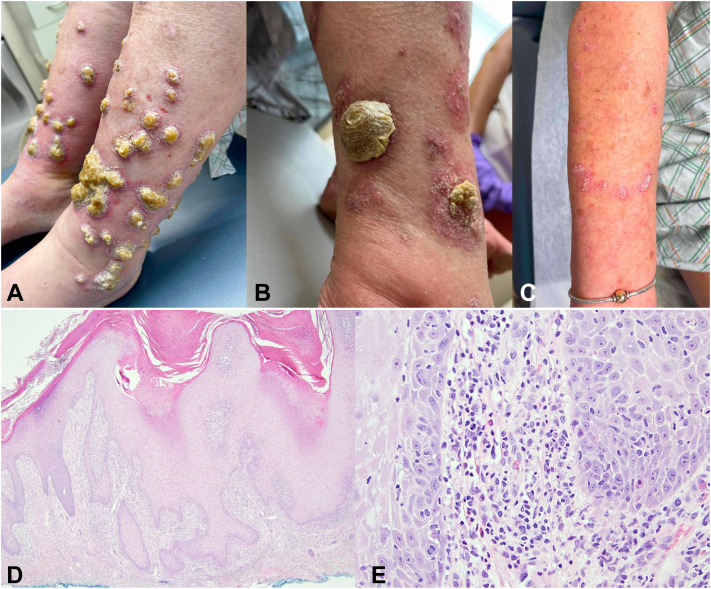
**(A)** Lesions initially presented on the bilateral lower extremities as pink plaques with white scale that progressed to form nodules with rock-hard yellow central crusts. **(B)** Heaped up ostraceous crust with tall cutaneous horn-like thickening. **(C)** New, pink scaly plaques emerging on forearm. **(D)** Florid pseudoepitheliomatous hyperplasia is noted on low magnification (hematoxylin and eosin, ×40). **(E)** Interface alteration with lymphocytic infiltration and scattered eosinophils are present (H&E, ´200).

Physical examination revealed hyperkeratotic, thickened plaques with rupioid and ostraceous scale morphologies, along with thick adherent yellow crust on the bilateral lower legs (Fig 1, *A* and *B*). There were also new pink, scaly plaques emerging on the forearms (Fig 1, *C*). There were no notable laboratory abnormalities. Subsequent biopsies from lesions on the right forearm and left lower leg were taken (Fig 1, *D* and *E*).

**Question 1: Which of the following is an appropriate next step in management for this condition?**

**A.** Potent topical corticosteroids
**B.** Topical imiquimod
**C.** Mohs micrographic surgery
**D.** Referral to plastic surgery
**E.** Oral acitretin

**Answer discussion:**

**E.** A diagnosis of hypertrophic lichen planus (HLP) was made. Initiation of systemic therapy with acitretin (initially 10 mg daily, increased to 20 mg daily) led to significant clinical improvement, evidenced by gradual detachment of crusts and residual flat pink inflammation. Intralesional triamcinolone injections further improved larger, more resistant lesions. After approximately 6 months, the patient successfully tapered off acitretin and used topical triamcinolone to manage residual inflammation with sustained clearance.

HLP is an idiopathic inflammatory variant of lichen planus, clinically characterized by hyperkeratotic nodules or plaques predominantly affecting the lower extremities of adults aged 50 to 75 years.^1^ Lesions are often pruritic and painful to touch, although they can be asymptomatic. HLP’s pathogenesis is thought to involve cytotoxic CD8^+^ T lymphocytes that mediate epidermal damage through the release of proinflammatory cytokines, including interferon gamma and tumor necrosis factor-α.^1^^,^^2^ The differential diagnosis includes a broad range of neoplastic, inflammatory, and infectious conditions, such as squamous cell carcinoma, hypertrophic actinic keratosis, keratoacanthoma, benign lichenoid keratosis, prurigo nodularis, hypertrophic lupus erythematosus, viral verrucae, halogenoderma, and deep fungal or mycobacterial infections.^1^ Due to the broad differential, clinicopathologic correlation is necessary for accurate diagnosis.

Notable biopsy features of HLP include pseudoepitheliomatous hyperplasia, hyperorthokeratosis, wedge-shaped hypergranulosis, and lymphocytic infiltrates concentrated at the tips of rete ridges, often accompanied by eosinophils.^1^ Treatment of HLP often begins with potent topical corticosteroids. For more extensive or treatment-refractory cases, intralesional corticosteroids, narrowband UV-B phototherapy, 5-fluorouracil, or systemic agents such as prednisolone, acitretin, methotrexate, mycophenolate mofetil, tofacitinib, or thalidomide may be considered.^1^^,^^3^^,^^4^ Although multiple options show efficacy, high-quality comparative studies remain limited.^4^ Proper diagnosis and treatment can prevent unnecessary surgical procedures for misdiagnoses of neoplastic lesions.^1^

This case underscores the importance of maintaining diagnostic flexibility and recognizing treatment nonresponse as a prompt to reevaluate prior assumptions; early consideration of HLP in recalcitrant lower extremity plaques may prevent unnecessary morbidity and guide more effective, targeted therapy.

## Conflicts of interest

None disclosed.
